# Estimating Cyanobacteria Community Dynamics and its Relationship with Environmental Factors

**DOI:** 10.3390/ijerph110101141

**Published:** 2014-01-20

**Authors:** Wenhuai Luo, Huirong Chen, Anping Lei, Jun Lu, Zhangli Hu

**Affiliations:** 1 Shenzhen Key Laboratory of Marine Bioresource and Eco-environmental Science, Shenzhen Engineering Laboratory of Marine Algal Biotechnology, College of Life Science, Shenzhen University, Shenzhen 518060, China; E-Mails: luowh127@gmail.com (W.L.); huirong.c@gmail.com (H.C.); bioaplei@szu.edu.cn (A.L.); jun.lu@aut.ac.nz (J.L.); 2 Institute for Applied Ecology New Zealand, School of Applied Sciences, and School of Interprofessional Health Studies, Faculty of Health and Environmental Sciences, and Institute of Biomedical Technology, Auckland University of Technology, 34 St Paul Street, Auckland 1142, New Zealand

**Keywords:** eutrophication, cyanobacteria community composition, PCR-DGGE, freshwater lakes

## Abstract

The cyanobacteria community dynamics in two eutrophic freshwater bodies (Tiegang Reservoir and Shiyan Reservoir) was studied with both a traditional microscopic counting method and a PCR-DGGE genotyping method. Results showed that cyanobacterium *Phormidium tenue* was the predominant species; twenty-six cyanobacteria species were identified in water samples collected from the two reservoirs, among which fourteen were identified with the morphological method and sixteen with the PCR-DGGE method*.* The cyanobacteria community composition analysis showed a seasonal fluctuation from July to December. The cyanobacteria population peaked in August in both reservoirs, with cell abundances of 3.78 × 10^8^ cells L^-1^ and 1.92 × 10^8^ cells L^-1^ in the Tiegang and Shiyan reservoirs, respectively. Canonical Correspondence Analysis (CCA) was applied to further investigate the correlation between cyanobacteria community dynamics and environmental factors. The result indicated that the cyanobacteria community dynamics was mostly correlated with pH, temperature and total nitrogen. This study demonstrated that data obtained from PCR-DGGE combined with a traditional morphological method could reflect cyanobacteria community dynamics and its correlation with environmental factors in eutrophic freshwater bodies.

## 1. Introduction

Eutrophication of water bodies and subsequent cyanobacteria blooms have become a worldwide environmental problem since last century. Toxins produced by some cyanobacteria species pose a threat to public health [1]. In China, a survey done in 2000 showed that around 37.8 % of its reservoirs were eutrophic, representing 13.4 % of total water supply capacity [2]. The situation is worse in Guangdong Province in South China. As shown in a survey done in 132 Guangdong reservoirs during 2002–2003, two reservoirs were hyper-eutrophic, 12 reservoirs were meso-eutrophic, and most studied reservoirs (111 out of 132) were eutrophic (total phosphorus concentration around 0.01 to 0.05 mg L^-1^) [3]. The city of Shenzhen is located in south Guangdong, and its tropical weather and fast economic development increase the chances of reservoir eutrophication and cyanobacteria blooms. It is necessary to develop a fast and reliable assessment method to evaluate the phytoplankton community composition and predict the occurrence of cyanobacteria blooms, which is of economic, health and environmental importance to Shenzhen City.

Shiyan Reservoir (longitude 99°8’ E, latitude 37°6’ N) is located in Shiyan Town, in the Bao’an District of Shenzhen. The mean water depth is 36.0 m and the capacity is 31,200,000 m^3^. Tiegang Reservoir (longitude 98°8’ E, latitude 30°0’ N) is located in Xixiang Town of Shenzhen. Its capacity is 68,400,000 m^3^. The two reservoirs are connected by an open channel. Shiyan Reservoir is the major urban water supply for Bao’an District, providing drinking water for surrounding towns since 1994 [4]. Water quality in both reservoirs was eutrophic [3,5] with visible algal blooms in some areas [4]. However, little study has been done on the phytoplankton community dynamics in these reservoirs. 

Currently the traditional morphological observation method using a light microscope is still commonly used to study the population dynamics of phytoplankton communities in eutrophic water bodies. It is time consuming and easily influenced by personal error. Some researchers also use high performance liquid chromatography methods to analyze toxic cyanobacteria blooms, but these methods needs commercial toxin standards, which are expensive and not easily available [6]. PCR- based denaturing gradient gel electrophoresis (DGGE) is now being used often in cyanobacteria ecology studies. The PCR-DGGE technique was invented to detect site mutations [7] and incorporated a microbial ecology method [8]. In the last decade, this technique has been used widely in environmental microorganism studies [9,10,11,12]. Worldwide cyanobacteria bloom events have attracted researchers to apply PCR-DGGE to study cyanobacteria community composition [13,14,15]. It is crucial to choose the most typical gene clusters for PCR amplification and subsequent DGGE analysis. The most commonly used gene sequences are conservative genes on rRNA, especially on 16S rRNA. As the intergenic transcribed spacer (ITS) region between 16S-23S rRNA gene is non-coding and variable, the ITS sequence has become more commonly used in this area [16,17,18]. In this study, we applied both an ITS-based PCR-DGGE method and the traditional morphological method to investigate the cyanobacteria communities in the Tiegang and Shiyan reservoirs of Shenzhen. We also used Canonical Correspondence Analysis (CCA) to study the relationship between cyanobacteria community dynamics and environmental factors.

## 2. Experimental Section

### 2.1. Sample Collection and Determination of Water Quality

In 2007, surface water samples were collected with a water sampler from the center and outlet of the Shiyan and Tiegang reservoirs at the beginning of each month. Center and outlet samples were combined to perform physical-chemical analysis. Transparency was measured with a Secchi disk. Dissolved oxygen (DO), pH, and temperature were measured in the field with a YSI ProPlus multiparmameter (YSI Inc., Yellow Springs, OH, USA). Chemical parameters including permanganate index (COD_Mn_), total nitrogen (TN), ammonia (NH_4_^+^-N) and total phosphorus (TP) were determined in the laboratory according to the National Environmental Quality Standards for Surface Water (GB3838-2002) [19]. Chlorophyll *a* concentration was measured using an ethanol extraction method modified from Lorenzen [20].

Phytoplankton samples were collected at the above-mentioned sampling sites and put into 1 L sample bottles. Lugol’s solution (15 mL) was added to each bottle, and set overnight. Supernatant was carefully removed, and the final concentrated sample volume was 50 mL. Each sample was vortexed and one drop of sample was placed on a haemocytometer to be examined under an Olympus-BX51 compound microscope (Olympus, Tokyo, Japan) with 400× magnification. For each sample, five fields in the haemocytometer were counted and the mean value was used to calculate the biomass. For colonies or filaments, only the parts within the fields were counted. The phytoplankton biomass was expressed as cell numbers per liter. For qualitative examination, phytoplankton net #25 (0.064-mm-diameter) tow samples fixed with formaldehyde solution (final concentration 5%) were put in counting chamber to identify genus or species of bacterium under inverted microscope (Olympus, Tokyo, Japan) [21]. 

### 2.2. DNA Extraction and PCR-DGGE Analysis

Water samples collected from Shiyan and Tiegang reservoirs during July and December 2007 were used for the ITS based PCR-DGGE analysis. Samples were first filtered through 0.45 μm filter paper and the filters were then used for DNA extraction with the Wizard Genomic DNA Purification Kit (Promega, Madison, WI, USA). PCR primers used for this study were CSIF/373R [22] that designed for ITS sequence of cyanobacteria genome. The sequences of primers were GC-CSIF (5′-G(T/C)C ACG CCC GAA GTC (G/A)TT AC-3′) and 373R(5′-CTA ACC ACC TGA GCT AAT-3′) with a 40 bp hairpin sequence on the 5′ (5′-CGC CCG CCG CGC CCC GCG CCC GGCCCG CCG CCC CCG CCC C-3′), size of the amplification sequence is around 250 bp.

PCR reactions were performed in microcentrifuge tube with total volume of 50 μL containing 8 μL of 10× buffer (with MgCl_2_), 1 μL each of reverse and forward primers, 8 μL of dNTP, 0.5 μL of *Taq* DNA polymerase, 28.5 μL of double distilled water, 5 μL of BSA, and 1μL of template DNA. Touchdown PCR amplification performed with 1 cycle of pre-denaturation at 94 °C for 5 min, 23 cycles of touchdown (94 °C for 40 s, 58–55 °C for 30 s with decreasing annealing temperature by 1 °C each consecutive cycle, 72 °C for 30 s), 26 cycles of amplification (94 °C for 40 s, 55 °C for 30 s and 72 °C for 30 s) and a final extension at 72 °C for 10 min. It was then incubate at 12 °C for 30 min.

DGGE was performed following the protocol provided in the manual for Bio-Rad DCode Universal Mutation Detection System (Bio-Rad Laboratories, Hercules, CA, USA). Denaturing gradient gel was 8% (wt/vol) polyacrylamide gels in 1× TAE buffer (20 mM Tris-acetate (pH 7.4), 10 mM acetate, 0.5 mM disodium EDTA). The gradient range was 25–45%. Electrophoresis was carried out at 50 V for 30 min and 120 V for 7 h. Gel was stained for 1 h with 3× GelRed TM Nucleic Acid Gel Stain (containing 0.1 M NaCl and 30 μL GelRed TM Nucleic Acid Gel Stain, 10,000× in water per100 mL H_2_O). Bands on gel were captured using gel image system. A band was considered to be a band when it provided a signal to noise ratio of over 3:1. After image capture, the gel plug containing a PCR product was removed with 10 μL pipette tips and placed in 1.5 mL microcentrifuge tube. The gel plug was then submerged in 50 μL of deionized water and sat at 4 °C overnight. Another DGGE was performed using excised band and original sample to verify the band. The next day, the solution was diluted 100× and 1 μL of the diluted extract was used for second PCR amplification (30 cycles, Ta = 57 °C). The PCR product was directly sequenced. When direct sequencing failed, sequencing was done after cloning with pUC57 T-vector system according to the manufacturer’s instructions (Takara, Dalian, China). Again, another DGGE was performed to verify the clone product by running the clone product with the original sample on one gel. The sequences were compared with GenBank database with BLAST search. Species was assigned based on the top BLAST hit. DGGE images were analyzed using software Quantity One (Bio-Rad). After recognition of each band, Un-weighted Pair Group Method with Arithmetic Averages (UPGMA) analysis was performed. Bands were also quantified and entered in Excel and used with physical-chemical indices in Canonical Correspondence Analysis (CCA) using CANOCO (version 4.5), as described in previously published reports [23,24]. 

## 3. Results and Discussion

### 3.1. Eutrophication Levels of Two Reservoirs

Tiegang Reservoir and Shiyan Reservoir are both important drinking water source for Shenzhen. The rapid economic development and continuous population growth have accelerated eutrophication in the two reservoirs during the last five years. In this study, nine water quality indices (TN, DO, NH_4_^+^-N, TP, COD_Mn_, pH, temperature, transparency and chlorophyll *a*) of both reservoirs were monitored monthly in 2007 and the mean values were shown in Table 1. 

**Table 1:** Mean value of water quality parameters in Tiegang and Shiyan Reservoirs in 2007 (standard deviations in parentheses).

Water Quality Parameters	Tiegang Reservoir	Shiyan Reservoir
Water temperature (°C)	25.4 (5.56)	24.9 (5.52)
DO (mg L^-1^)	8.38 (1.26)	8.16 (1.42)
Chlorophyll *a* (μg L^-1^)	45.3 (31.2)	53.0 (26.9)
COD_Mn_ (mg L^-1^)	2.75 (0.745)	3.04 (0.674)
Ammonia (mg L^-1^)	0.147 (0.087)	0.566 (0.359)
Total nitrogen (mg L^-1^)	0.934 (0.242)	1.508 (0.387)
Total phosphorus (mg L^-1^)	0.034 (0.009)	0.043 (0.004)
pH	8.237 (0.566)	7.871 (0.657)
Transparency (cm)	64.8 (5.59)	58.3 (6.68)

### 3.2. Phytoplankton and Cyanobacteria Community Structure and Dynamics in Two Reservoirs

**Figure 1:**
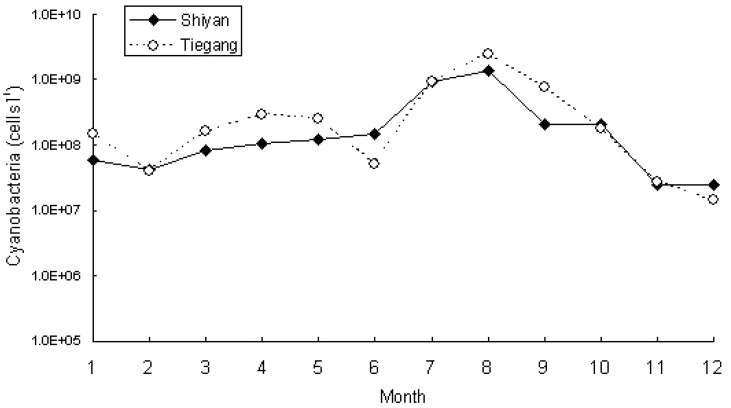
The annual changes of cyanobacteria abundance in Tiegang and Shiyan Reservoir.

Cyanobacteria, green algae (*Scenedesmus* sp. and *Cosmarium* sp.) and diatoms (*Synedra* spp, *Melosira* spp.) were the main phytoplankton groups in the tested water samples. Cyanobacteria were the most dominant phytoplankton in Tiegang Reservoir and were also abundant in Shiyan Reservoir, except for the winter, during which diatoms were dominant. The cyanobacterium *Phormidium tenue* was found consistently in all of the water samples, and other common cyanobacterial species including *Raphidiopsis sinensia* and species belonging to *Chroococcales* sp. and *Merismopedia* sp. Cyanobacteria abundance varied monthly. Winter showed the lowest cell density, with 1.40 × 10^7^ cells L^-1^ in December for Tiegang and 2.50 × 10^7^ cells L^-1^ for Shiyan (Figure 1). The highest phytoplankton cell density appeared in August where 2.48 × 10^9^ cells L^-1^ and 1.39 × 10^9^ cells L^-1^ were found in samples collected from Tiegang and Shiyan, respectively. For the rest of the year, the cyanobacteria abundance was around 10^8^ cells L^-1^ in both reservoirs. As the cyanobacteria abundance did not vary much from January to June (Figure 1), we only used samples from July to December to analyze the cyanobacteria abundance and population composition with both the traditional microscopic counting method and a PCR-DGGE genotyping method. Results from microscopic investigation are listed in Table 2.

**Table 2:** Monthly abundance of main cyanobacteria species in Tiegang and Shiyan Reservoirs from July to December, 2007.

Cyanobacteria species	T7	T8	T9	T10	T11	T12	S7	S8	S9	S10	S11	S12
*Phormidium tenue*	1.2 × 10^8^	4.8 × 10^8^	2.1 × 10^8^	5.4× 10^7^	1.2 × 10^7^	9.0 × 10^6^	3.0 × 10^7^	1.2 × 10^8^	2.7 × 10^7^	8.4 × 10^7^	6.0 × 10^6^	3.0 × 10^6^
*Raphidiopsis sinensia*	7.5 × 10^7^	5.3 × 10^8^	4.4 × 10^8^	8.3 × 10^7^	N	5.0 × 10^6^	2.0 × 10^8^	2.8 × 10^8^	1.1 × 10^8^	5.0 × 10^7^	2.5 × 10^6^	1.0 × 10^7^
*Microcystis aeruginosa*	N	N	3.8 × 10^7^		N	N	2.5 × 10^7^	N	2.0 × 10^7^	5.0 × 10^6^	N	N
*Chroococcus giganteus*	N	N	2.5 × 10^7^	N	N	N	N	N	3.8 × 10^7^	N	N	N
*Chroococcus westii*	N	1.3 × 10^8^	N	N	N	N	N	6.5 × 10^7^	N	N	N	8.0 × 10^6^
*Chroococcus limneticus*	N	N	N	1.6 × 10^7^	N	N	N	N	N	3.8 × 10^7^	N	N
*Cylindrospermum* sp*.*	4.3 × 10^8^	1.3 × 10^9^	N	N	N	N	4.5 × 10^8^	6.8 × 10^8^	N	N	N	N
*Spirunila major*	N	N	N	N	N	N	N	N	N	N	N	N

T1–T6: Samples from July to December in Tiegang Reservoir; S1–S6: Samples from July to December in Shiyan Reservoir; N means not detectable, cell numbers <5.0 × 10^5^cells L^-1^.

**Figure 2:**
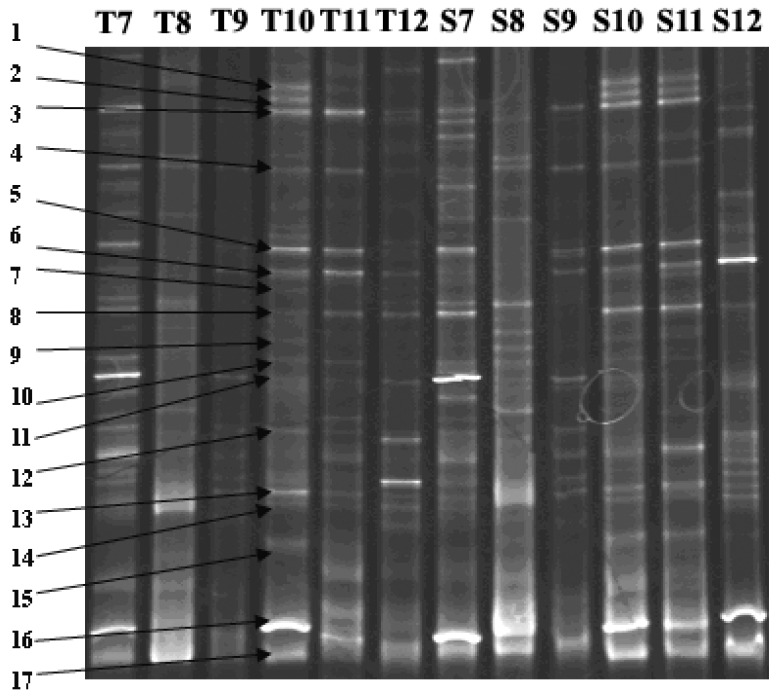
The PCR-DGGE fingerprint map of water samples from July to December 2007 in Tiegang and Shiyan Reservoir. T7-T12: samples from July to December in Tiegang Reservoir; S7-S12: samples from July to December in Shiyan Reservoir.

**Figure A1:**
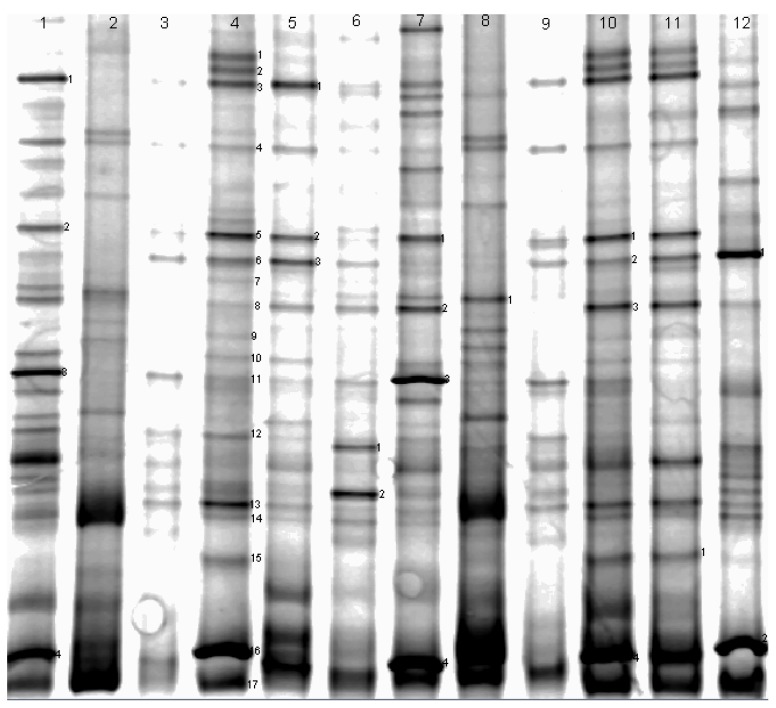
Larger PCR-DGGE fingerprint map for sequencing. Lanes 1-6: samples from July to December in Tiegang Reservoir; Lanes 7-12: samples from July to December in Shiyan Reservoir.

Figure 2 shows PCR-DGGE results of water samples collected from Tiegang and Shiyan Reservoirs from July to December (more details are shown in Figure A1 and Table A1 and Table A2 in Appendix). As summarized in Table 3 (e-value of each comparison was under 0.001), 16 cyanobacteria genotypes corresponding to 16 species were identified in each reservoir, including *Microcystis*, *Phormidium, Synechocystis, Cylindrospermopsis, Spirulina, Arthrospira, Raphidiopsis, Lynghya* and *Anabeana*. For these 16 species, each species had one specific band, except for *Cylindrospermopsis raciborskii* (bands 11 and 13) (Table 3). The brightness of the band was used as an indicator of cyanobacteria density. For example, band 16 in Figure 2 was very bright, and the corresponding *Phormidium* sp. was also shown to be dominant genera under microscope investigation (Table 2). However, it should be noted that the PCR step could favor the amplification of particular DNA segments, which may cause an underestimation of certain strains of bacteria. In the current study, the comparison of dominant species between PCR-DGGE and microscopic analyses seemed to be compatible. 

**Figure 3:**
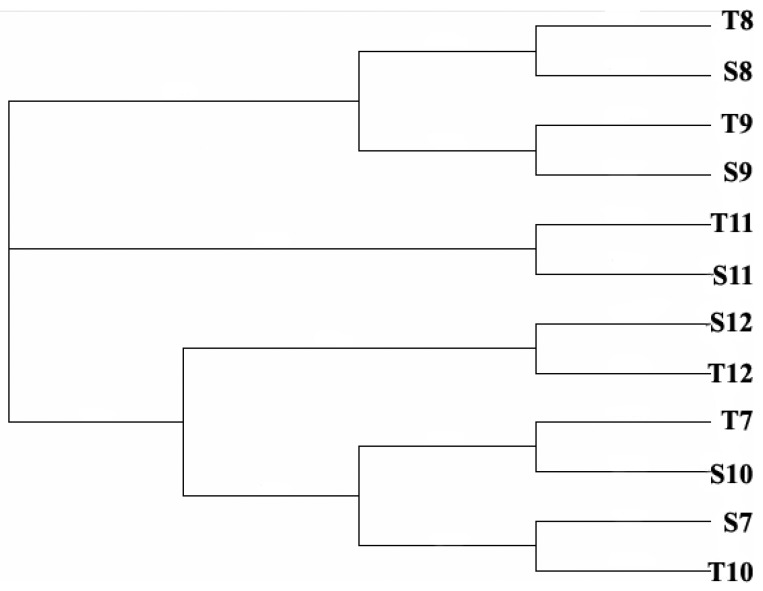
The cyanobacteria community structure system tree map of Tiegang and Shiyan reservoir water samples from July to December in 2007. T7–T12: Samples from July to December in Tiegang Reservoir; S7–S12: Samples from July to December in Shiyan Reservoir. The purpose of the tree is to show the clades.

Band numbers of DGGE products were compared among samples using Quantity One (Bio-Rad). T10 was designated as the standard for relative quantification. Bands at the same position were considered as the same species. The relative biomass was represented by the DNA amounts from the bands. The Cs (Dice coefficient) correlation between relative biomass of each band ranged from 38.1% (T8 and S12) to 78.8% (T11 and S11), which means cyanobacteria community in December of Shiyan and August of Tiegang were mostly different, while the two reservoirs had similar cyanobacteria communities in November. Based on similarity analysis, results were converted into UPGMA diagram (Figure 3) using Quantity One. The tree had three major clades. Clade I consisted of cyanobacteria species in August and September (Lanes T8, S8, T9 and S9). Clade II consisted of cyanobacteria species in November (Lanes T11, S11). Clade III consisted of samples collected in December (Lanes T12, S12), October and July (Lanes T7, S10, S7, and T10). Overall, the cyanobacteria community structure was very similar between the two reservoirs in the same month while it showed seasonal changes in the same reservoir. 

**Table 3:** The sequencing result of bands in Figure 2.

DGGE Band No.	Similarity Number	Closest Matching Organism	Base Pairs Compared	Similarity (%)
1	AF363949.1	*Microcoleus steenstrupii*	171	81
2	EF583859.1	*Anabaena* sp.	139	97
3	X75045.1	*Spirulina* sp.	130	92
4	AM398947.1	*Phormidium* sp.	222	97
5	EF583859.1	*Anabaena* sp.	150	98
6	AJ605201.1	*Microcystis* sp.	244	98
7	EF150986.1	*Microcystis* sp.	214	97
8	EU183353.1	*Arthrospira* sp.	204	94
9	DQ351315.1	*Synechococcus* sp. UW140	209	91
10	AM398973.1	*Phormidium* sp	211	96
11	AM502073.1	*Cylindrospermopsis raciborskii*	346	98
12	DQ786166.1	*Leptolyngbya* sp. LLi18	145	94
13	AJ582284.1	*Cylindrospermopsis raciborskii*	379	94
14	BA000022.2	*Synechocystis* sp	158	89
15	X75045.1	*Spirulina* sp	130	92
16	AM398960.1	*Phormidium persicinum* SAG 80.79	135	98
17	DQ351315.1	*Synechococcus* sp. UW140 16S	209	91

### 3.3. Relationship between Cyanobacteria Community Dynamics and Environment Factors

**Figure 4:**
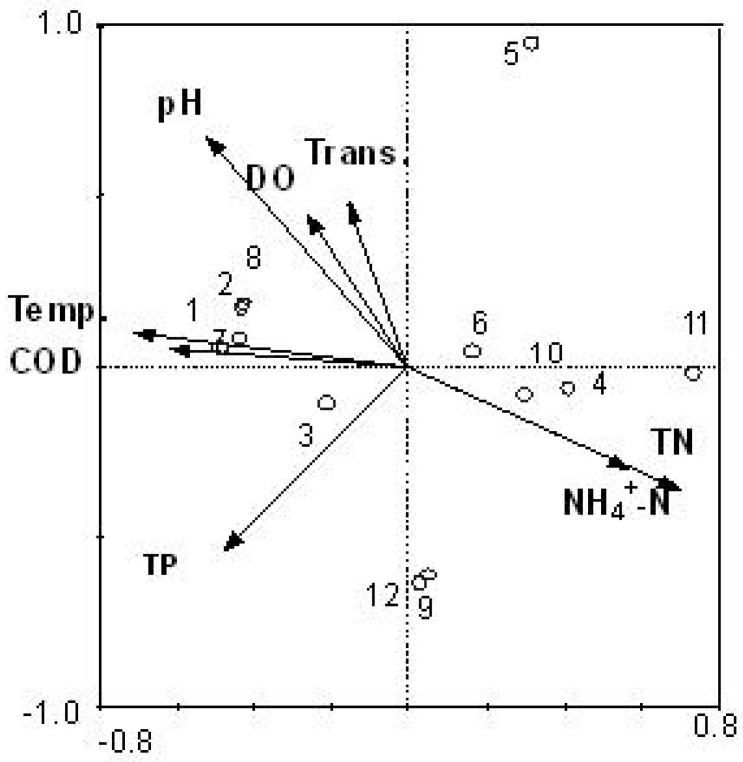
Canonical correspondence analysis (CCA) ordination diagram of the cyanobacteria community dynamics data (from traditional morphological method) in relation to the environmental variables. 1–6: samples from July to December in Tiegang Reservoir: 7–12: samples from July to December in Shiyan Reservoir.

The cell number of each cyanobacteria species in water samples of two reservoirs was counted under a microscope. These numbers were analyzed for correlations with environmental factors using CCA. Results are shown in Figure 4. The cyanobacteria community structure correlated mainly with temperature, pH, COD, NH_4_^+^-N and TN, with coefficients around 0.7.

**Figure 5:**
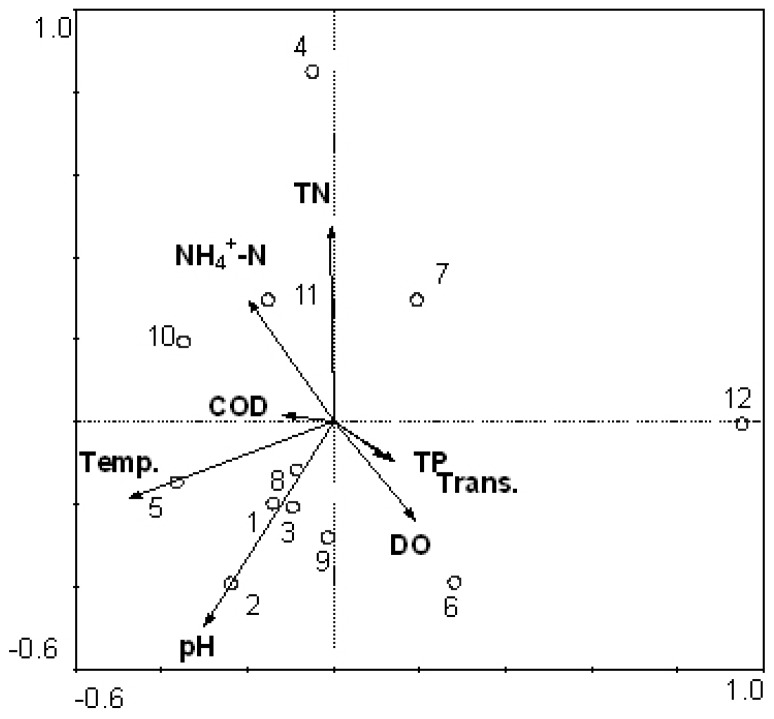
CCA ordination diagram of the cyanobacteria community dynamics data (from PCR-DGGE approach) in relation to the environmental variables. 1–6: samples from July to December in Tiegang Reservoir: 7–12: samples from July to December in Shiyan Reservoir.

The number of bands and their relative quantities from PCR-DGGE results were also analyzed for correlation with environmental factors using CCA. Results are shown in Figure 5. The cyanobacteria community dynamics in the two reservoirs were mainly correlated with temperature, pH, and TN (*R* > 0.5). Results from both methods indicated that temperature, pH, and TN are important factors affecting cyanobacteria community structure, which is consistent with other reports that those are the main parameters for cyanobacterial growth [25,26]. This result is also in line with previous data from other reservoirs [27]. The increase in *Microcystic aeruginosa* and *Phormidium tenue* is an important indication of eutrophication [28]. It is necessary to monitor cyanobacteria community dynamics of reservoirs, and study its relationship with the environmental factors for the estimation and evaluation of eutrophication level of water bodies. Either or both of the methods employed in this study can serve as a useful environmental monitoring tool, and the correlation between cyanobacteria community and environmental factors can be used to predict and prevent cyanobacteria bloom. 

### 3.4. Comparison between Morphological Identification and PCR-Dgge Identification to Determine Cyanobacteria Community of Two Reservoirs

This study employed both microscopic observation and PCR-DGGE analysis to identify cyanobacteria species in water bodies and compared the results. In this particular study, it was found that the number of cyanobacteria species observed in PCR-DGGE was much larger than the number of species identified by microscopy. In October 2007, for example, five species were identified by the microscopic method in Tiegang Reservoir (Table 2, T10); while sixteen species were identified by PCR-DGGE analysis in the same sample (Figure 2, T10). The cyanobacteria community of two reservoirs depicted in Figure 2 (data from PCR-DGGE analysis) also showed better diversity than in Table 2 (data from microscopic observation) in other months of 2007. When comparing Table 2 and Table 3, we can see the main cyanobacteria species identified were also different. Band 5 in Table 2, for example, was identifies as *Anabaena* sp. and detected in most samples (Figure 2), while no *Anabaena* was found through microscopic method (Table 2). *Chroococcus* sp., on the other hand, was found in many samples with high density in Table 2, but no band in PCR-DGGE was identified as *Chroococcus* sp. Both methods have their disadvantage and may cause false results. Microscopic analysis requires professional experience and skills for morphological identification, and it is prone to human error. For example, *Synechococcus* spp. is a very small unicelluar genera and the biomass could probably be overestimated under microscope. For PCR-DGGE, the primer set (CSIF/373R) used in this study was good for broadly scan dominated cyanobacteria isolates, but different cyanobacteria isolates might show as a same band on the gel [22]. However, the most dominated cyanobacteria genera were consistently identified as *Phormidium* sp. through both methods, which indicated that PCR-DGGE could objectively reflect main cyanobacteria community dynamics compared with morphological identification. Pyrosequencing is another tool to perform similar analysis. With the steady decrease of the cost, this technique may be an alternative or complementary tool for environmental analysis, such as the one described here. It will certainly improve the reliability of the data. 

### 3.5. Reliability of CCA Based on PCR-DGGE Data

In most microbiology studies, it is common to use relative quantity data of PCR-DGGE bands to perform CCA [24,29,30]. However, it is not always possible to confirm the correlation between the relative quantity of DNA bands with the exact number of bacteria because large number of bacteria exists in water samples and not all of them could be isolated and identified with morphological methods. It is relatively easier to quantify and identify cyanobacteria species with morphological methods, so in this study we used data from both PCR-DGGE analysis and a morphological method to perform CCA in relation to environmental factors. This provides a good chance to check the reliability of CCA based on PCR-DGGE data of cyanobacteria. Results suggested that the cyanobacteria community dynamics determined by traditional morphological method showed better correlation coefficients with temperature, pH, TN and other environmental factors, such as COD and NH_4_^+^-N (Figure 4). Results of CCA from PCR-DGGE data was largely in accordance with Figure 4 in terms of the correlation with temperature and TN. However, there were also obvious differences when comparing Figure 4 with Figure 5. For example, CCA results from PCR-DGGE could not identify the close correlation between cyanobacteria community and COD and NH_4_^+^-N. The lower correlation coefficient from PCR-DGGE data might be due to the DNA band intensity cannot accurately reflect the quantity of the relevant species. Moreover, the sample distribution in the CCA analysis was also different (Figure 4 and Figure 5). In general, the relative quantification of cyanobacteria with PCR-DGGE method using CSIF/373R primers can be applied in CCA as a reference tool to seek the correlation with environmental factors of water bodies in reservoir. However, results need be calibrated and verified by traditional morphological methods.

## 4. Conclusions

We investigated the cyanobacteria community composition in eutrophic water samples with both the PCR-DGGE method and the traditional microscopic examination method. Both methods provided useful information and most results were comparable. Both reservoirs were dominated with cyanobacteria during the summer months, with temperature, precipitation, TN and pH as the main factors correlated with cyanobacteria abundance. As a tool to study cyanobacteria communities, PCR-DGGE does have its drawbacks, for example, no primers could amplify specific DNA bands from all cyanobacterial species, and cyanobacteria DNA sequences in GenBank are limited. Currently, PCR-DGGE analysis can be used as a semi-quantitative tool to identify algal species, and with the combination of traditional morphological methods, it could effectively monitor community dynamics of cyanobacteria in reservoirs.

## Appendix

**Table A1:** List of sequencing results from DGGE bands on Figure A1.

DGGE band no.	similarity number	closest matching organism	base pairs compared	similarity(%)
1-1	X75045.1	*Spirulina* sp.	130	92
1-2	AM398960.1	*Phormidium persicinum* SAG 80.79	135	98
2-1	BA000022.2	*Synechocystis* sp.	158	89
4-1	AF363949.1	*Microcoleus steenstrupii*	171	81
4-2	EF583859.1	*Anabaena* sp.	139	97
4-3	X75045.1	*Spirulina* sp.	130	92
4-4	AM398947.1	*Phormidium* sp.	222	97
4-5	EF583859.1	*Anabaena* sp.	150	98
4-6	AJ605201.1	*Microcystis* sp.	244	98
4-7	EF150986.1	*Microcystis* sp.	214	97
4-8	EU183353.1	*Arthrospira* sp.	204	94
4-9	DQ351315.1	*Synechococcus* sp. UW140	209	91
4-10	AM398973.1	*Phormidium* sp.	211	96
4-11	AM502073.1	*Cylindrospermopsis raciborskii*	346	98
4-12	DQ786166.1	*Leptolyngbya* sp. LLi18	145	94
4-13	AJ582284.1	*Cylindrospermopsis raciborskii*	379	94
4-14	BA000022.2	*Synechocystis* sp.	158	89
4-15	X75045.1	*Spirulina* sp.	130	92
4-16	AM398960.1	*Phormidium persicinum* SAG 80.79	135	98
4-17	DQ351315.1	*Synechococcus* sp. UW140 16S	209	91
5-1	EU183353.1	*Arthrospira* sp.	204	94
5-2	EF583859.1	*Anabaena* sp.	150	98
5-3	EF150986.1	*Microcystis* sp.	214	97
6-1	AY672727.1	*Microcystis* sp.	394	98
6-2	AJ582275.1	*Raphidiopsis* sp.	368	96
7-1	EF583859.1	*Anabaena* sp.	150	98
7-2	EU183353.1	*Arthrospira* sp.	204	94
7-3	AM502073.1	*Cylindrospermopsis raciborskii*	220	98
7-4	AM398960.1	*Phormidium persicinum*	135	98
8-1	EF442201.1	*Synechococcus* sp.	89.8	92
10-1	EF583859.1	*Anabaena* sp.	150	98
10-2	EF150986.1	*Microcystis* sp.	214	97
10-3	EU183353.1	*Arthrospira* sp.	204	94
10-4	AM398960.1	*Phormidium persicinum*	135	98
11-1	EF429298.1	*Leptolyngbya badia*	130	98
12-1	EF150986.1	*Microcystis* sp.	214	97
12-2	AM398960.1	*Phormidium persicinum* SAG	135	98

**Table A2:** List of DNA sequences of bands in Table A1.

DGGE Band No.	Similarity Number	Closest Matching Organism	Base Pairs Compared	Similarity (%)
1-1	X75045.1	*Spirulina* sp.	130	92
CCCGTTACGCTGCGACGAATGCGTGGCTAGATGACAGGGGTGAGTCGTAACAAGGTAGCCGTACCGGAAGGTGTGGCTGGATCACCTCCTTTAAGGGAGACCGATGACAGATAGTGTACGAATGAATGTAAGCTATCAGTTGGTCATCTCAAGGTCGAGGGTTTCGAGTATGGTATTCTTCAGGCTAGGGTCTAGGGGCTATTAGCTCAGGTGGTTAGA				
1-2	AM398960.1	*Phormidium persicinum* SAG 80.79	135	98
TTCCCTCAGGGGGGGGTGCGACGCAGGTCTGATGACTGGGGTGAAGTCGTAACAAGGTAGCCGTACCGGAAGGTGTGGCTGGATCACCTCCTTTAAGGGAGACCGATGACGGATAGTTTACGAATAGATGTAAGGTATCAGTTGGTCATCTCGAGGTCGAGGGTTGGGAGTATGGTATTCTTCAGGCTAGGGTCTAGGGGCTATTAGCTAGGTGGTTAGA				
2-1	BA000022.2	*Synechocystis* sp.	158	89
CGGATAGGAAGGAAGAGCTAACGTAGGACTGATGACTGGGGTGAGTCGTAACAAGGTAGCCGTACCGGAAGGTGTGGCTGGATCACCTCCTTTTAGGGAGACCTAATCCACTTAGAAATGTTAAGGAAACTACCATAACAACCTAAATTGGTCTAACCTAGGTCGGTCGCAGACTTGAAGTAAGTCTTTCAAACTATGATTTGGTTCGATAAGGGCTATTAACTCAGGTGGTTAGA				
4-2	EF583859.1	*Anabaena* sp.	139	97
TTTTTGGGGGAGGCGCGACGCACGCTGATGACTGGGGTGAGTCGTAACAAGGTAGCCGTACCGGAAGGTGTGGCTGGATCACCTCCTTTTAGGGAGACCCAATCCGTAGAAGTTATGAGTTATGAGTTTTGAATGTTGAGTTTAAGACTTGTGACCTAAATCTAAACATTACAACTTCTATGAGATTCAATCCCGAGGTCGTACCGAGGTTGTGAACTTTCAAGCTAAGTCAGGTTTGTAAATGGGCTATTAGCTCAGGTGGTTAGA				
4-3	X75045.1	*Spirulina* sp.	130	92
CCCGTTACGCTGCGACGAATGCGTGGCTAGATGACAGGGGTGAGTCGTAACAAGGTAGCCGTACCGGAAGGTGTGGCTGGATCACCTCCTTTAAGGGAGACCGATGACAGATAGTGTACGAATGAATGTAAGCTATCAGTTGGTCATCTCAAGGTCGAGGGTTTCGAGTATGGTATTCTTCAGGCTAGGGTCTAGGGGCTATTAGCTCAGGTGGTTAGA				
4-4	AM398947.1	*Phormidium* sp.	222	97
TTCCCTCAGGGGGGGGTGCGACGCAGGTCTGATGACTGGGGTGAAGTCGTAACAAGGTAGCCGTACCGGAAGGTGTGGCTGGATCACCTCCTTTAAGGGAGACCGATGACGGATAGTTTACGAATAGATGTAAGGTATCAGTTGGTCATCTCGAGGTCGAGGGTTGGGAGTATGGTATTCTTCAGGCTAGGGTCTAGGGGCTATTAGCTAGGTGGTTAGA				
4-5	EF583859.1	*Anabaena* sp.	150	98
TTTTTGGGGGAGGCGCGACGCACGCTGATGACTGGGGTGAGTCGTAACAAGGTAGCCGTACCGGAAGGTGTGGCTGGATCACCTCCTTTTAGGGAGACCCAATCCGTAGAAGTTATGAGTTATGAGTTTTGAATGTTGAGTTTAAGACTTGTGACCTAAATCTAAACATTACAACTTCTATGAGATTCAATCCCGAGGTCGTACCGAGGTTGTGAACTTTCAAGCTAAGTCAGGTTTGTAAATGGGCTATTAGCTCAGGTGGTTAGA				
4-6	AJ605201.1	*Microcystis* sp.	244	98
CCAGTAGGGAGGGGGAGCTAGTAGGACTGGTGACTGGGGTGAGTCGTACAAGGTAGCCGTACCGGAAGGTGTGGCTGGATCACCTCCTTTTAGGGAGACCTACCCATTGAAGAATCGAAAGCCGAAGGCGAATAGAGAATCAAATGGTCTACTCTAGGTCGATGACGTGAGATTGTGAAGTCTTTCAAACTAATATTTGGTTCGCGGGCTATTAGCTATGTGGTTAGA				
4-7	EF150986.1	*Microcystis* sp.	214	97
CCGTAGCCAAGGGAGAGCTAGCATGACTGATGACTGGGGTGAAGTCGTAACAAGGTAGCCGTACCGGAAGGTGTGGCTGGATCACCTCCTTTCAGGGAGACCTTACCCACCTCAACTCCAAAGCACAAAGCGAATAGAGAGAGGATTGGTCAACCTAAGTCGGTCGAGGAATTGTGTGGCTCTCAAACTTGTCTGGGTTTACTTCTAAGAAGAAGGGAAACGAGGGCTATTAGCTAAGGTGGTTAGAGACATTACCTCAGGTGGTTAGA				
4-8	EU183353.1	*Arthrospira* sp.	204	94
AGGATCCGAATCAGGTCTTTTATGACCCCAGAACCTAGTTTGAAAGCCACATACCTCGTTCCGACCTTTTGGGATTGATTCTTGGTTTCGACTACTATTTTTTCGTCTTATACCCGAATTAGGTCTCCCTTTAAGGAGGTGATCCAGCCACACCTTCCGGTACGGCTACCTTGTTACGACTTCACCCCAGTCACTAGCCCTGCCTTAGGCATCCCCCTCCTTGCGGTTGAGGTAACGACTTCGGGCGTGACA				
4-9	DQ351315.1	*Synechococcus* sp. UW140	209	91
CAATGAAGAGAGAGCGTATGTGGGGCTGATGACTGGGGTGAGTCGTAACAAGGTAGCCGTACCGGAAGGTGCGGCTGGATCACCTCCTAACAGGGAGACACAACTGATTTTGATGTTTGGTTCATTTTGAAATCAAGCCGAAATCCTGTCACCTTAGGTCGATCGGTACCTCAGATGGTTGAATGCAATGGGAGCGGAAACGCGACCAAAGCATCTGCCACCTCAGTTCCTAAACTTCTGTCTAGGTCACCCCTCCGAGCCCATCTGGGCCATTAGCTCAGGTGGTTAGA				
4-10	AM398973.1	*Phormidium* sp.	211	96
ACATTAAAGGGTAGAGCGACGCACGCTGATGACTGGGGTGAGTCGTAACAAGGTAGCCGTACCGGAAGGTGTGGCTGGATCACCTCCTTTAAGGGAGACCGATGACAGATAGTGTACGAATGAATGTAAGCTATCAGTTGGTCATCTCAAGGTCGAGGGTTTCGAGTATGGTATTCTTCAGGCTAGGGTCTAGGGGCTATTAGCTAGGTGGTTAGA				
4-11	AM502073.1	*Cylindrospermopsis raciborskii*	346	98
CGTAAGGTAGCAGCCGATAGCGCGAGTAGAGACTAGACGTGAGTCGTAACAAGGTAGCCGTACCGGAAGGTGTGGCTGGATCACCTCCTTTTAGGGAGACCTACCCATTGAAGAATCCAAAGCCGCAGGCGAATAGAGAATCAAATGGTCTACTCTAGGTCGATGACGTGAGATTGTGAAGTCTTTCAAACTAATATTTGGTTCGCGGGCTATTAGCTCAGGTGGTTAGAACACACCATGGGACCAGACCTTGTCCAAGACCCCTTTTGCTTTACTTAATGACAAAAAACAAAGATCTACCAAACTTTTTACCCAATAAAAATATCCCGGGTCCCCAGCACCCCTTGTTCCCTCAAAAATTTCCCCAAAAAAACCCGACCCCCCTATTATCTCAAAGCGCTTCCTTTTGTTGGGGATGGGGGACAAAAATTGGGGGGGCCACACAAAGTGATCTTATAGTGCCCTCTGGCTTTTATCTGGGGCATCGGAAAACTCTTAATTCTGTATCGGACCTCCACGCTCGTGTCTTTGGGGGGGGCTACCATATCGAGAGAACTCTCCGCATGCGGAGCTCTCTCTACAGTGCGCGGGGGTT				
4-12	DQ786166.1	*Leptolyngbya* sp. LLi18	145	94
CCGTAGCCAAGGGAGAGCTAGCATGACTGATGACTGGGGTGAAGTCGTAACAAGGTAGCCGTACCGGAAGGTGTGGCTGGATCACCTCCTTTCAGGGAGACCTTACCCACCTCAACTCCAAAGCACAAAGCGAATAGAGAGAGGATTGGTCAACCTAAGTCGGTCGAGGAATTGTGTGGCTCTCAAACTTGTCTGGGTTTACTTCTAAGAAGAAGGGAAACGAGGGCTATTAGCTAAGGTGGTTAGAGACATTACCTCAGGTGGTTAGA				
4-13	AJ582284.1	*Cylindrospermopsis raciborskii*	379	94
CCCATCAGTGAGCTATGTAGGACTGGTGACTGGGGTGAGTCGTAACAAGGTAGCCGTACCGGAAGGTGTGGCTGGATCACCTCCTTTTAGGGAGACCTACCCATTGAGGAATCGAAAGCGGAGAGCGAATAGAGAATCAAATGGTCTACTCTAGGTCGGTGACGTGAGATTGTGAAGTCTTTCAAACTAATATTTGGTTCGCGAGAGGGCTATTAGCTAGGGTGGTTAGAAGCACCCCCGGGGGATAGCCAACCACTGCGGGCTTAAACCCTGGGGAAAAAACCAAAGTGGTAAGAACAGCTGGGGGCAAAAAAATAATCAAGACTCCGAATTTCCTGTGTTCCCTCAAAAATTTCTTTGAGAACCACCGACCCCCCTGTATATCTGACTGCCGCTCTTTGCCGATCTTTTTTTTAAAATGGTGGCCGGCCCCCCAAATGATGTGTTGTTGGCGCCCCCCCCCTCTTACTTGGCGTTCGAGAGAATTACTAATACGACATTCATCCACCACGGTTTTATTTAGTGGGGGGCGCGAACGGAGAGATGGCT				
4-14	BA000022.2	*Synechocystis* sp.	158	89
CGGATAGGAAGGAAGAGCTAACGTAGGACTGATGACTGGGGTGAGTCGTAACAAGGTAGCCGTACCGGAAGGTGTGGCTGGATCACCTCCTTTTAGGGAGACCTAATCCACTTAGAAATGTTAAGGAAACTACCATAACAACCTAAATTGGTCTAACCTAGGTCGGTCGCAGACTTGAAGTAAGTCTTTCAAACTATGATTTGGTTCGATAAGGGCTATTAACTCAGGTGGTTAGA				
4-15	X75045.1	*Spirulina* sp.	130	92
CCCGTTACGCTGCGACGAATGCGTGGCTAGATGACAGGGGTGAGTCGTAACAAGGTAGCCGTACCGGAAGGTGTGGCTGGATCACCTCCTTTAAGGGAGACCGATGACAGATAGTGTACGAATGAATGTAAGCTATCAGTTGGTCATCTCAAGGTCGAGGGTTTCGAGTATGGTATTCTTCAGGCTAGGGTCTAGGGGCTATTAGCTCAGGTGGTTAGA				
4-16	AM398960.1	*Phormidium persicinum* SAG 80.79	135	98
TTCCCTCAGGGGGGGGTGCGACGCAGGTCTGATGACTGGGGTGAAGTCGTAACAAGGTAGCCGTACCGGAAGGTGTGGCTGGATCACCTCCTTTAAGGGAGACCGATGACGGATAGTTTACGAATAGATGTAAGGTATCAGTTGGTCATCTCGAGGTCGAGGGTTGGGAGTATGGTATTCTTCAGGCTAGGGTCTAGGGGCTATTAGCTAGGTGGTTAGA				
4-17	DQ351315.1	*Synechococcus* sp. UW140 16S	209	91
CAATGAAGAGAGAGCGTATGTGGGGCTGATGACTGGGGTGAGTCGTAACAAGGTAGCCGTACCGGAAGGTGCGGCTGGATCACCTCCTAACAGGGAGACACAACTGATTTTGATGTTTGGTTCATTTTGAAATCAAGCCGAAATCCTGTCACCTTAGGTCGATCGGTACCTCAGATGGTTGAATGCAATGGGAGCGGAAACGCGACCAAAGCATCTGCCACCTCAGTTCCTAAACTTCTGTCTAGGTCACCCCTCCGAGCCCATCTGGGCCATTAGCTCAGGTGGTTAGA				
5-1	EU183353.1	*Arthrospira* sp.	204	94
AGGATCCGAATCAGGTCTTTTATGACCCCAGAACCTAGTTTGAAAGCCACATACCTCGTTCCGACCTTTTGGGATTGATTCTTGGTTTCGACTACTATTTTTTCGTCTTATACCCGAATTAGGTCTCCCTTTAAGGAGGTGATCCAGCCACACCTTCCGGTACGGCTACCTTGTTACGACTTCACCCCAGTCACTAGCCCTGCCTTAGGCATCCCCCTCCTTGCGGTTGAGGTAACGACTTCGGGCGTGACA				
5-2	EF583859.1	*Anabaena* sp.	150	98
TTTTTGGGGGAGGCGCGACGCACGCTGATGACTGGGGTGAGTCGTAACAAGGTAGCCGTACCGGAAGGTGTGGCTGGATCACCTCCTTTTAGGGAGACCCAATCCGTAGAAGTTATGAGTTATGAGTTTTGAATGTTGAGTTTAAGACTTGTGACCTAAATCTAAACATTACAACTTCTATGAGATTCAATCCCGAGGTCGTACCGAGGTTGTGAACTTTCAAGCTAAGTCAGGTTTGTAAATGGGCTATTAGCTCAGGTGGTTAGA				
5-3	EF150986.1	*Microcystis* sp.	214	97
CCGTAGCCAAGGGAGAGCTAGCATGACTGATGACTGGGGTGAAGTCGTAACAAGGTAGCCGTACCGGAAGGTGTGGCTGGATCACCTCCTTTCAGGGAGACCTTACCCACCTCAACTCCAAAGCACAAAGCGAATAGAGAGAGGATTGGTCAACCTAAGTCGGTCGAGGAATTGTGTGGCTCTCAAACTTGTCTGGGTTTACTTCTAAGAAGAAGGGAAACGAGGGCTATTAGCTAAGGTGGTTAGAGACATTACCTCAGGTGGTTAGA				
6-1	AY672727.1	*Microcystis* sp.	394	98
TCGCCAGTCGAGGTATCCATGCGCGTACTAGTGATGGGGTGCAGTCGTAACAAGGTAGCCGTACCGGAAGGTGTGGCTGGATCACCTCCTTAAAGGGAGACCTAATTCAGGTAGGATACGAAAAAAAGTAGTCCCTACCAAGAATCAATCCCAAAAGGTCGGAGCGAGGCAAAATTGGCTTTCAAACTAGGTTCTGGGTTCACATAAGACCTGAATCAGGAACAAGGGCTATTAGCTCAGGTGGTTAGAATTAACTCCTGGGGTAGTTGGATCCAAGGTGGTTAGATTACCGCGGGTGTATGGGTTTCTAAAGATTCATTAACGAAGTTCAGGTTCAGCTTCTGTGCCCAAAGACTGAATGTAATTACTTGCAGACTCTGACGATATTTTCCCCAGAACTTTACCTTTGGGGTTTTTTTTTTGTCCTAAAACGAGCTCCATATGATCAGGGGGGGGGGGGACCCCCCCCCACCTCGTTCTCCCGGTCCACTCTCATCCATGATGAACCCCACGGACCAATCATACTCAGTCTGGATATAATGCATCAAAATACTCATGATTCTCTGGTTTCTCGGTACCACCTGGTACCTCTCGCACCCC				
6-2	AJ582275.1	*Raphidiopsis* sp.	368	96
CCCATCAGTGAGCTATGTAGGACTGGTGACTGGGGTGAGTCGTAACAAGGTAGCCGTACCGGAAGGTGTGGCTGGATCACCTCCTTTTAGGGAGACCTACCCATTGAGGAATCGAAAGCGGAGAGCGAATAGAGAATCAAATGGTCTACTCTAGGTCGGTGACGTGAGATTGTGAAGTCTTTCAAACTAATATTTGGTTCGCGAGAGGGCTATTAGCTAGGGTGGTTAGAAGCACCCCCGGGGGATAGCCAACCACTGCGGGCTTAAACCCTGGGGAAAAAACCAAAGTGGTAAGAACAGCTGGGGGCAAAAAAATAATCAAGACTCCGAATTTCCTGTGTTCCCTCAAAAATTTCTTTGAGAACCACCGACCCCCCTGTATATCTGACTGCCGCTCTTTGCCGATCTTTTTTTTAAAATGGTGGCCGGCCCCCCAAATGATGTGTTGTTGGCGCCCCCCCCCTCTTACTTGGCGTTCGAGAGAATTACTAATACGACATTCATCCACCACGGTTTTATTTAGTGGGGGGCGCGAACGGAGAGATGGCT				
7-1	EF583859.1	*Anabaena* sp.	150	98
TTTTTGGGGGAGGCGCGACGCACGCTGATGACTGGGGTGAGTCGTAACAAGGTAGCCGTACCGGAAGGTGTGGCTGGATCACCTCCTTTTAGGGAGACCCAATCCGTAGAAGTTATGAGTTATGAGTTTTGAATGTTGAGTTTAAGACTTGTGACCTAAATCTAAACATTACAACTTCTATGAGATTCAATCCCGAGGTCGTACCGAGGTTGTGAACTTTCAAGCTAAGTCAGGTTTGTAAATGGGCTATTAGCTCAGGTGGTTAGA				
7-2	EU183353.1	*Arthrospira* sp.	204	94
AGGATCCGAATCAGGTCTTTTATGACCCCAGAACCTAGTTTGAAAGCCACATACCTCGTTCCGACCTTTTGGGATTGATTCTTGGTTTCGACTACTATTTTTTCGTCTTATACCCGAATTAGGTCTCCCTTTAAGGAGGTGATCCAGCCACACCTTCCGGTACGGCTACCTTGTTACGACTTCACCCCAGTCACTAGCCCTGCCTTAGGCATCCCCCTCCTTGCGGTTGAGGTAACGACTTCGGGCGTGACA				
7-3	AM502073.1	*Cylindrospermopsis raciborskii*	220	98
CGTAAGGTAGCAGCCGATAGCGCGAGTAGAGACTAGACGTGAGTCGTAACAAGGTAGCCGTACCGGAAGGTGTGGCTGGATCACCTCCTTTTAGGGAGACCTACCCATTGAAGAATCCAAAGCCGCAGGCGAATAGAGAATCAAATGGTCTACTCTAGGTCGATGACGTGAGATTGTGAAGTCTTTCAAACTAATATTTGGTTCGCGGGCTATTAGCTCAGGTGGTTAGAACACACCATGGGACCAGACCTTGTCCAAGACCCCTTTTGCTTTACTTAATGACAAAAAACAAAGATCTACCAAACTTTTTACCCAATAAAAATATCCCGGGTCCCCAGCACCCCTTGTTCCCTCAAAAATTTCCCCAAAAAAACCCGACCCCCCTATTATCTCAAAGCGCTTCCTTTTGTTGGGGATGGGGGACAAAAATTGGGGGGGCCACACAAAGTGATCTTATAGTGCCCTCTGGCTTTTATCTGGGGCATCGGAAAACTCTTAATTCTGTATCGGACCTCCACGCTCGTGTCTTTGGGGGGGGCTACCATATCGAGAGAACTCTCCGCATGCGGAGCTCTCTCTACAGTGCGCGGGGGTT				
7-4	AM398960.1	*Phormidium persicinum*	135	98
TTCCCTCAGGGGGGGGTGCGACGCAGGTCTGATGACTGGGGTGAAGTCGTAACAAGGTAGCCGTACCGGAAGGTGTGGCTGGATCACCTCCTTTAAGGGAGACCGATGACGGATAGTTTACGAATAGATGTAAGGTATCAGTTGGTCATCTCGAGGTCGAGGGTTGGGAGTATGGTATTCTTCAGGCTAGGGTCTAGGGGCTATTAGCTAGGTGGTTAGA				
8-1	EF442201.1	*Synechococcus* sp.	89.8	92
CAATGAAGAGAGAGCGTATGTGGGGCTGATGACTGGGGTGAGTCGTAACAAGGTAGCCGTACCGGAAGGTGCGGCTGGATCACCTCCTAACAGGGAGACACAACTGATTTTGATGTTTGGTTCATTTTGAAATCAAGCCGAAATCCTGTCACCTTAGGTCGATCGGTACCTCAGATGGTTGAATGCAATGGGAGCGGAAACGCGACCAAAGCATCTGCCACCTCAGTTCCTAAACTTCTGTCTAGGTCACCCCTCCGAGCCCATCTGGGCCATTAGCTCAGGTGGTTAGA				
10-1	EF583859.1	*Anabaena* sp.	150	98
TTTTTGGGGGAGGCGCGACGCACGCTGATGACTGGGGTGAGTCGTAACAAGGTAGCCGTACCGGAAGGTGTGGCTGGATCACCTCCTTTTAGGGAGACCCAATCCGTAGAAGTTATGAGTTATGAGTTTTGAATGTTGAGTTTAAGACTTGTGACCTAAATCTAAACATTACAACTTCTATGAGATTCAATCCCGAGGTCGTACCGAGGTTGTGAACTTTCAAGCTAAGTCAGGTTTGTAAATGGGCTATTAGCTCAGGTGGTTAGA				
10-2	EF150986.1	*Microcystis* sp.	214	97
CCGTAGCCAAGGGAGAGCTAGCATGACTGATGACTGGGGTGAAGTCGTAACAAGGTAGCCGTACCGGAAGGTGTGGCTGGATCACCTCCTTTCAGGGAGACCTTACCCACCTCAACTCCAAAGCACAAAGCGAATAGAGAGAGGATTGGTCAACCTAAGTCGGTCGAGGAATTGTGTGGCTCTCAAACTTGTCTGGGTTTACTTCTAAGAAGAAGGGAAACGAGGGCTATTAGCTAAGGTGGTTAGAGACATTACCTCAGGTGGTTAGA				
10-3	EU183353.1	*Arthrospira* sp.	204	94
AGGATCCGAATCAGGTCTTTTATGACCCCAGAACCTAGTTTGAAAGCCACATACCTCGTTCCGACCTTTTGGGATTGATTCTTGGTTTCGACTACTATTTTTTCGTCTTATACCCGAATTAGGTCTCCCTTTAAGGAGGTGATCCAGCCACACCTTCCGGTACGGCTACCTTGTTACGACTTCACCCCAGTCACTAGCCCTGCCTTAGGCATCCCCCTCCTTGCGGTTGAGGTAACGACTTCGGGCGTGACA				
10-4	AM398960.1	*Phormidium persicinum*	135	
TTCCCTCAGGGGGGGGTGCGACGCAGGTCTGATGACTGGGGTGAAGTCGTAACAAGGTAGCCGTACCGGAAGGTGTGGCTGGATCACCTCCTTTAAGGGAGACCGATGACGGATAGTTTACGAATAGATGTAAGGTATCAGTTGGTCATCTCGAGGTCGAGGGTTGGGAGTATGGTATTCTTCAGGCTAGGGTCTAGGGGCTATTAGCTAGGTGGTTAGA				
11-1	EF429298.1	*Leptolyngbya badia*	130	98
GACTTTACGGCAGAGCGTCGCATGCTGATGACTGGGGTGAGTCGTAACAAGGTAGCCGTACCGGAAGGTGTGGCTGGATCACCTCCTTTAAGGGAGACCGATGACGGATAGTTTACGAATAGATGTAAGGTATCAGTTGGTCATCTCGAGGTCGAGGGTTGGGAGTATGGTATTCTTCAGGCTAGGGTCTAGGGGCTATTAGCTAGGTGGTTAGA				
12-1	EF150986.1	*Microcystis* sp.	214	
CCGTAGCCAAGGGAGAGCTAGCATGACTGATGACTGGGGTGAAGTCGTAACAAGGTAGCCGTACCGGAAGGTGTGGCTGGATCACCTCCTTTCAGGGAGACCTTACCCACCTCAACTCCAAAGCACAAAGCGAATAGAGAGAGGATTGGTCAACCTAAGTCGGTCGAGGAATTGTGTGGCTCTCAAACTTGTCTGGGTTTACTTCTAAGAAGAAGGGAAACGAGGGCTATTAGCTAAGGTGGTTAGAGACATTACCTCAGGTGGTTAGA				
12-2	AM398960.1	*Phormidium persicinum* SAG	135	
TTCCCTCAGGGGGGGGTGCGACGCAGGTCTGATGACTGGGGTGAAGTCGTAACAAGGTAGCCGTACCGGAAGGTGTGGCTGGATCACCTCCTTTAAGGGAGACCGATGACGGATAGTTTACGAATAGATGTAAGGTATCAGTTGGTCATCTCGAGGTCGAGGGTTGGGAGTATGGTATTCTTCAGGCTAGGGTCTAGGGGCTATTAGCTAGGTGGTTAGA				
